# Astrocytic metabolic and inflammatory changes as a function of age

**DOI:** 10.1111/acel.12268

**Published:** 2014-09-19

**Authors:** Tianyi Jiang, Enrique Cadenas

**Affiliations:** 1 Pharmacology and Pharmaceutical Sciences, School of Pharmacy, University of Southern CaliforniaLos Angeles, CA, 90089-9121, USA

**Keywords:** astrocytes, cytokines, hydrogen peroxide, inflammation, mitochondria, mitochondrial biogenesis, nitric oxide, NFκB

## Abstract

This study examines age-dependent metabolic-inflammatory axis in primary astrocytes isolated from brain cortices of 7-, 13-, and 18-month-old Sprague–Dawley male rats. Astrocytes showed an age-dependent increase in mitochondrial oxidative metabolism respiring on glucose and/or pyruvate substrates; this increase in mitochondrial oxidative metabolism was accompanied by increases in COX3/18SrDNA values, thus suggesting an enhanced mitochondrial biogenesis. Enhanced mitochondrial respiration in astrocytes limits the substrate supply from astrocytes to neurons; this may be viewed as an adaptive mechanism to altered cellular inflammatory–redox environment with age. These metabolic changes were associated with an age-dependent increase in hydrogen peroxide generation (largely ascribed to an enhanced expression of NOX2) and NFκB signaling in the cytosol as well as its translocation to the nucleus. Astrocytes also displayed augmented responses with age to inflammatory cytokines, IL-1β, and TNFα. Activation of NFκB signaling resulted in increased expression of nitric oxide synthase 2 (inducible nitric oxide synthase), leading to elevated nitric oxide production. IL-1β and TNFα treatment stimulated mitochondrial oxidative metabolism and mitochondrial biogenesis in astrocytes. It may be surmised that increased mitochondrial aerobic metabolism and inflammatory responses are interconnected and support the functionality switch of astrocytes, from neurotrophic to neurotoxic with age.

## Introduction

Brain aging is accompanied by a hypometabolic state that involves decreased glucose uptake, reduced expression and translocation to the plasma membrane of neuronal glucose transporters GLUT3 and GLUT4, and an imbalance of insulin (the PI3K/Akt pathway)- and c-Jun N-terminal kinase (JNK) signaling; these changes impinge on mitochondrial metabolism and neuronal survival (Jiang *et al*., 2013; Yin *et al*., 2013). Astrocytes have a supportive function for neurons in the central nervous system: Neurons harbor a strong aerobic metabolism, while astrocytes primarily rely on the ATP derived from glycolysis with lactate extrusion as the end point (Dienel & Hertz, 2001; Bolaños *et al*., 2010). Lactate diffuses from astrocytes and is taken up subsequently by neurons through high-affinity monocarboxylate transporter 2 (MCT2) to serve as a key metabolite for neuronal aerobic metabolism and thus meeting the large energy demands in neuronal activity (Magistretti, 2011; Suzuki *et al*., 2011). Age-dependent glial fibrillary acidic protein (GFAP, a marker of astrocytes) expression and astrocyte activation have been reported, but little is known about the astrocytic metabolic state, especially because astrocyte activation requires energy. An *in vivo* magnetic resonance spectroscopy study revealed reduced neuronal mitochondrial metabolism and increased glial mitochondrial metabolism with age in human brains (Boumezbeur *et al*., 2010).

Astrocytes are intimately involved in age-dependent inflammatory responses (Campuzano *et al*., 2009): They sense and amplify inflammatory signals from microglia and initiate a series of inflammatory responses that involve NFκB activation and the genes under its control (Zhang *et al*., 2013). The transcription factor NFκB is redox-sensitive, that is, responsive to H_2_O_2_ modulation, and is also stimulated by proinflammatory cytokines such as TNFα and IL-1β (Li & Verma, 2002). Persistent activation of NFκB engages MAPK activation (Guma *et al*., 2011), and stress-responsive JNK signaling was especially involved in insulin resistance and inflammation (Han *et al*., 2013) and in aging and neurodegenerative diseases (Mehan *et al*., 2011; Jiang *et al*., 2013). Astrocytes surround neurons and synapses, and the question arises on how the inflammatory responses generated by astrocytes propagate to neurons and affect their functions. Cytokines themselves and diffusible-redox species, such as H_2_O_2_ and nitric oxide are intercellular signals that impinge on neuronal function. Excessive amount of nitric oxide [•NO) produced by upregulation of inducible nitric oxide synthase (iNOS) was implicated in several central nervous system (CNS) disorders (Brosnan *et al*., 1994; Luth *et al*., 2001), and iNOS expression was controlled by NFκB signaling and JNK signaling (Wang *et al*., 2004). NADPH oxidase (NOX) enzymes are widely expressed in different cell types in the CNS and are major sources of oxidants (Sorce & Krause, 2009). H_2_O_2_ derived from NOX, especially NOX2, fulfills some physiologic functions such as host defense and cellular signaling, but excessive oxidative stress also contributes to chronic inflammation in aging and various neurodegenerative disorders (Park *et al*., 2007). Taking into account of their diffusibility, it may be surmised that •NO and H_2_O_2_ could act as intercellular mediators released from astrocytes and compromise neuronal functions in aging.

Because of the tight association between astrocytes and neurons in structural proximity, metabolic coupling and inflammatory responses (Belanger & Magistretti, 2009), it is intriguing to hypothesize that they switch from being neurotrophic to neurotoxic in aging and neurodegenerative diseases. Primary astrocytes from senescence-accelerated mouse (SAM) showed elevated oxidative stress and reduced neuroprotective capacity (Garcia-Matas *et al*., 2008). This study is aimed at exploring the mechanistic basis of the age-dependent metabolic-inflammatory axis in astrocytes. This was assessed on primary astrocytes derived from brain cortices of rats of different ages in terms of the age-dependent cellular metabolic shifts that are associated with inflammatory responses and how inflammatory cytokines modulate energy metabolism.

## Results

### Age-dependent astrocytic metabolic shift

The purity of the astrocyte preparation was assessed by GFAP (an astrocyte-specific intermediate filament) staining: Cultures were >99% GFAP positive, while staining for OX-42, a microglial marker, was negative (Fig.1). This indicates the purity of the astrocyte preparation free of contamination with microglia and is consistent with reports from other groups using the same isolation procedure, in which astrocyte cultures contained >98% astrocytes (Morgan *et al*., 1995).

**Figure 1:**
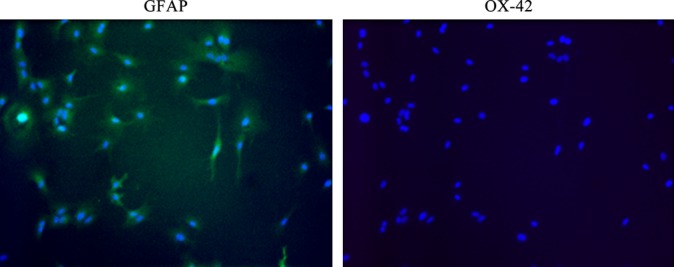
Purity of primary astrocytes. GFAP (green) and OX-42 (red) were used as markers for astrocytes and microglia, respectively. Nuclei were stained with DAPI. OX-42 staining was negative, thus indicating the lack of contamination with microglia.

Astrocytes mainly rely on anaerobic glycolysis for energy, and its aerobic (mitochondrial metabolism of pyruvate) is generally much weaker than that of neurons, which rely almost entirely on aerobic glycolysis. As mentioned above, lactate generated during anaerobic glycolysis in astrocytes supports neuronal oxidative metabolism, that is, oxidation of lactate to pyruvate and its further mitochondrial metabolism to satisfy neuronal energy demands (Magistretti, 2011; Suzuki *et al*., 2011).

Astrocyte metabolic profiles were assessed with the Extracellular Flux analyzer: Astrocytes isolated from 7-, 13-, and 18-month-old Sprague–Dawley rat cortices displayed an age-dependent increase in oxygen consumption rate (OCR) reflecting mitochondrial respiration (Table1; Fig.2). extracellular acidification rate (ECAR), reflecting anaerobic glycolysis (*i.e*., lactate formation) did not change with age (not shown). Figure2A shows a time course of astrocytes respiring on pyruvate (OCR): Basal respiration in astrocytes from 18-month-old rat (192 ± 9 pmoles min^−1^) cortices was ∼50% higher than that in astrocytes from 7-month-old cortices (130 ± 6 pmoles min^−1^); ATP turnover (following oligomycin addition) was also higher in the astrocytes from older rats. The most striking increase (∼two fold) was in maximal respiratory capacity (following the addition of FCCP); this resulted in a much higher reserve capacity (OCR_MAXIMAL RESPIRATORY CAPACITY_ − OCR_BASAL RESPIRATION_) (238 ± 29 pmoles min^−1^) in astrocytes from 18-month-old rat cortices (Fig.2A, Table1). Similar trends in maximal respiratory capacity and reserve capacity were observed with astrocytes respiring on glucose and glucose + pyruvate (Table1). The increased astrocytic aerobic metabolism may be a consequence of augmented mitochondrial biogenesis, as indicated by the increasing COX3/18SrDNA values with age (Fig.2B) (COX3 and 18SrDNA represent mitochondrial and nuclear genomes, respectively). Tetramethylrhodamine methyl ester (TMRM) staining (Fig.2C) revealed 35% and 51% increases in mitochondrial membrane potential of astrocytes from 13- and 18-month-old rats compared with astrocytes from 7-month-old rats (Fig.2D). TMRM staining cannot distinguish whether or not the increased membrane potential is related to increased mitochondria number.

**Table 1:** Bioenergetic parameters of primary astrocytes

	OCR (pmoles min^−1^)		
	Age (months)		
	7	13	18
Pyruvate			
Basal respiration	130 ± 6	122 ± 5	192 ± 9*
OXPHOS-induced respiration	72 ± 6	85 ± 3*	97 ± 9*
H+ leak-induced respiration	58 ± 11	36 ± 6	96 ± 8
Maximal respiratory capacity	224 ± 18	307 ± 9*	430 ± 21*
Nonmitochondrial respiration	42 ± 3	35 ± 2	71 ± 6
Reserve capacity	94 ± 11	185 ± 2*	238 ± 29*
Glucose			
Basal respiration	108 ± 11	113 ± 4	117 ± 12
OXPHOS-induced respiration	69 ± 7	79 ± 5	83 ± 7
H+ leak-induced respiration	40 ± 4	34 ± 2	34 ± 11
Maximal respiratory capacity	184 ± 23	226 ± 11*	266 ± 20*
Nonmitochondrial respiration	31 ± 9	34 ± 1	16 ± 12
Reserve capacity	76 ± 11	113 ± 5*	150 ± 9*
Glucose + Pyruvate			
Basal respiration	206 ± 6	263 ± 4*	222 ± 6
OXPHOS-induced respiration	139 ± 8	176 ± 7*	154 ± 7
H+ leak-induced respiration	68 ± 2	86 ± 4	68 ± 1
Maximal respiratory capacity	398 ± 11	503 ± 18*	532 ± 5*
Nonmitochondrial respiration	48 ± 8	59 ± 10	31 ± 10
Reserve capacity	191 ± 17	240 ± 21*	310 ± 10*

* *P *<* *0.05.

**Figure 2:**
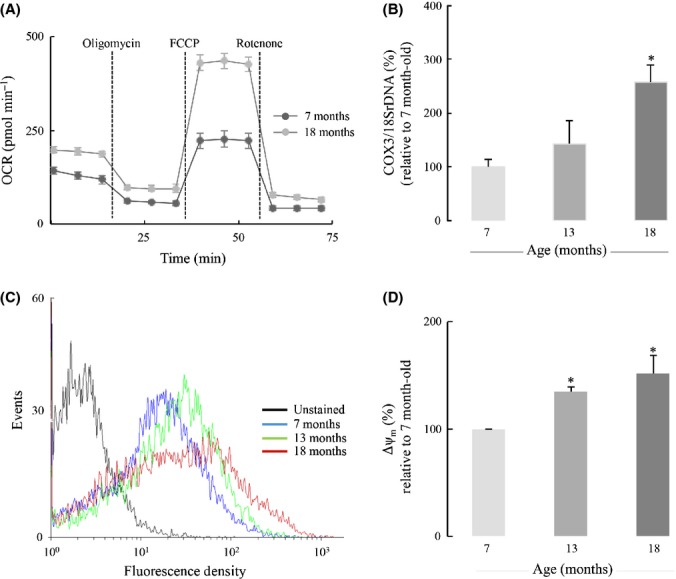
Age-dependent increases in astrocytic aerobic metabolism. (A) Representative figure of changes in astrocytic aerobic metabolism with age. Primary astrocytes were cultured on Seahorse XF-24 plates, and oxygen consumption rate (OCR) was measured in the presence of pyruvate as the solely important substrate. A more comprehensive and quantitative comparison of astrocytic aerobic metabolism in the presence of different substrates was shown in Table1. (B) Age-dependent increase in mitochondrial biogenesis was determined by the ratio of COX3/18SrDNA, assayed by real-time PCR. (C) Age-dependent increase in mitochondrial membrane potential was determined by TMRM staining using FACS. (D) Quantification of the increase in mitochondrial membrane potential with age. *P <* *0.05.

### Age-dependent increases in astrocytic H_2_O_2_ generation and NOX expression

Effective cellular sources of H_2_O_2_ are the electron leak of the mitochondrial respiratory chain, inflammatory and noninflammatory NADPH oxidases, and monoamine oxidase associated with the outer mitochondrial membrane. While the latter generates directly H_2_O_2_ upon the two-electron reduction of O_2_, the mitochondrial respiratory chain and NADPH oxidases generate H_2_O_2_ upon disproportionation of 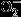. The rate of H_2_O_2_ release from primary astrocytes increased with age (Fig.3A): There was a 50% increase in the H_2_O_2_ release rate by astrocytes isolated from 13-month-old and 18-month-old rats relative to astrocytes from 7-month-old rats. To further investigate the sources of increased H_2_O_2_ release, primary astrocytes from different aged rats were treated with mitochondrial respiratory chain inhibitor antimycin A, monoamine oxidase (MAO) inhibitor deprenyl, and NOX inhibitor diphenyleneiodonium (DPI) (Fig.3A). H_2_O_2_ release from astrocytes was insensitive to antimycin A and deprenyl, whereas treatment with diphenyleneiodonium elicited a 50% decrease in H_2_O_2_ release rate; hence, the reduced H_2_O_2_ release rate was defined DPI-sensitive H_2_O_2_ release. The substantial increase in DPI-sensitive H_2_O_2_ release with age was most prominent in astrocytes from 13-month-old rats, with a 79% increase compared to astrocytes from 7-month-old rats (Fig.3B). This indicates that the increase in H_2_O_2_ release from older astrocytes was likely due to increased NOX activity. NOX2 and NOX4 are the two main isoforms of NOX in astrocytes, the former induced by inflammation and the latter is usually considered the constitutive NOX isoform. The DPI-sensitive H_2_O_2_ formation was associated with an age-dependent increase in NOX2 expression (60% and 100% increases in astrocytes from 13-month-old and 18-month-old rats, respectively) (Fig.3C). NOX4 expression slightly increased with age, especially in astrocytes from 13-month-old rats, despite no statistically significance (Fig.3D). Of note, data in Fig.2D show an age-dependent increase in mitochondrial membrane potential: This condition is always associated with a decreased efflux of H_2_O_2_ from mitochondria, and thus, it strengthens the notion that NOX activity is the major source of H_2_O_2_ in astrocytes and that it increases with age.

**Figure 3:**
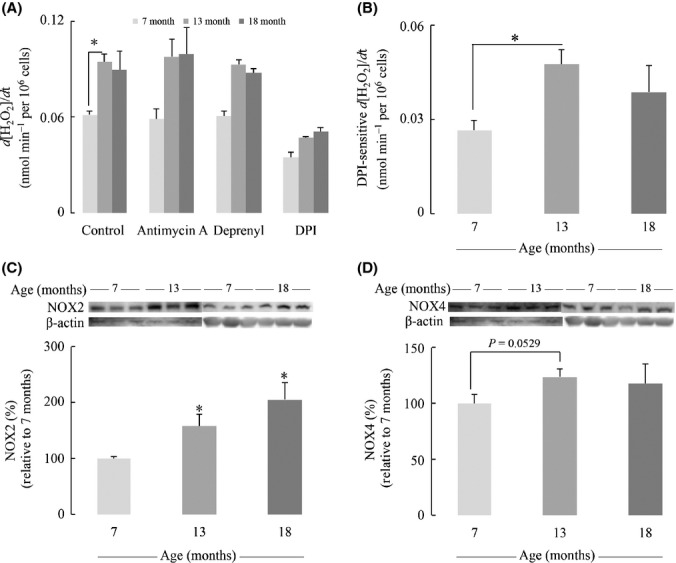
Age-dependent increases in Astrocytic H_2_O_2_ generation and NOX expression. (A) H_2_O_2_ release rate in the astrocytic medium was determined by Amplex Red assay. Primary astrocytes were treated with antimycin A, deprenyl, or DPI at 50 μm for 1 h to determine the contribution to H_2_O_2_ generation from different subcellular sources. (B) Increased DPI-sensitive H_2_O_2_ release rate with age. DPI-sensitive H_2_O_2_ release rate was defined by subtraction of H_2_O_2_ release rate with DPI treatment from the basal release rate. Expressions of (C) NOX2 and (D) NOX4 were determined by Western blot. *P <* *0.05.

### Age-dependent changes in the redox-sensitive NFκB transcription factor

NFκB is a redox-sensitive transcription factor; in addition to receptor engagement and phosphorylation of the IKK complex, the latter phosphorylates IκB with the consequent release of free NFκB and its translocation to the nuclei. Hence, NFκB remains sequestered in the cytosol by IκBα under basal conditions and translocates to the nucleus upon stimulation such as LPS, TNFα, and IL-1β, where it induces expression of genes involved in inflammatory responses such as iNOS, TNFα, and IL-1β. H_2_O_2_ promotes NFκB activation at least at two levels: H_2_O_2_ enhances phosphorylation of the IKK complex (either via stimulation of protein kinase D or inhibition of the phosphatase PP2A); H_2_O_2_ enhances the phosphorylation of IκB at Tyr^42^ and serine phosphorylation of p65, thus facilitating the dissociation of NFκB (Storz & Toker, 2003; Takada *et al*., 2003; Storz *et al*., 2004; Loukili *et al*., 2010).

The basal levels of NFκB in the cytosol of astrocytes from older rats were over twofold of those from young rats (Fig.4A). Upon stimulation with IL-1β, IκBα is rapidly degraded, thus resulting in free NFκB. Therefore, the ratio of cytosolic IκBα/NFκB was used to assess the sensitivity of the cytosolic NFκB pathway, with lower values indicating more available NFκB for translocation to the nucleus: Data in Fig.4B indicated that astrocytes isolated from 13-month-old and 18-month-old rats expressed a better responsiveness to IL-1β induction, reflected by a substantial decrease in IκBα/NFκB ratio compared with 7-month-old astrocytes (50% and 48% decrease compared with 73%). Constitutive NFκB levels in the nucleus of astrocytes increased with age, with 36% increase at 13-month-old and 150% increase at 18-month-old compared with those from 7-month-old rats (Fig.4C,D). Upon IL-1β treatment, astrocytes displayed robust increase in NFκB nuclear translocation with astrocytes from older rats showing even larger increase of NFκB in the nuclear fraction as compared with younger rats (56% increase at 13-month-old and more than threefold of expression at 18-month-old) (Fig.4C,D). Treatment with TNFα elicited similar trends but with more robust changes, that is*,* higher than those elicited by IL-1β and several fold higher than the constitutive NFκB nuclear levels (Fig.4C,D).

**Figure 4:**
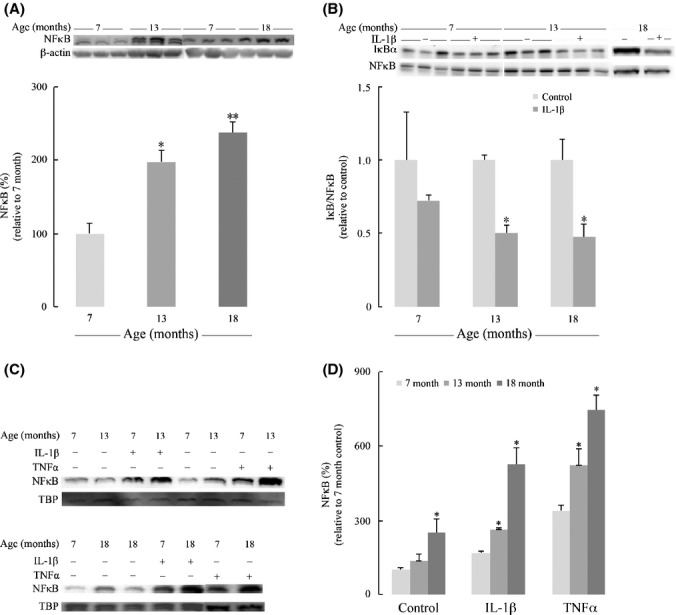
Age-dependent activation of NFκB pathway. (A) Age-dependent increase in cytosolic NFκB p65 level. (B) Age-dependent increases in the sensitivity of cytosolic NFκB signaling in response to 100 ng mL^−1^ IL-1β treatment. The sensitivity was determined by the ratio of IκBα and NFκB level indicating the freely available NFκB. (C) Age-dependent increases in NFκB p65 in the nuclear fractions of primary astrocytes at basal level and in response to 100 ng mL^−1^ IL-1β and 50 ng mL^−1^ TNFα treatments. TATA box-binding protein (TBP) was used as a loading control for nuclear fraction. (D) Quantification of NFκB p65 in the nuclear fractions. *P <* *0.05, **P <* *0.01.

### IL-1β induces activation of iNOS and JNK/p38 pathways in astrocytes – Effect of age

Inducible nitric oxide synthase (iNOS; NOS2) is largely responsible for inflammation-related long-lasting production of nitric oxide (•NO). Inducible nitric oxide synthase expression was hardly detectable under basal conditions but substantially increased in response to IL-1β treatment: Astrocytes from 13-month-old and 18-month-old rats showed stronger iNOS expression than younger counterparts (Fig.5A). Accordingly, •NO basal generation (measured as 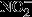 in the medium) in astrocytes from older rats was ∼40% higher than that in astrocytes from 7-month-old rats (Fig.5B). However, increased expression of iNOS upon treatment of astrocytes with IL-1β resulted in a large increase in 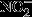 formation, with values 2.0- and 2.4-fold higher in astrocytes from older rats than those from 7-month-old rats (Fig.5B). Of note, treatment with IL-1β elicited a minor increase (47%) in 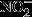 levels in astrocytes from 7-month-old rats (Fig.5B). The increased expression of iNOS and, as a corollary, of •NO generation is apparently at odds with the higher metabolic rates (OCR data in Table1) observed in astrocytes as a function of age, especially considering that a major function of •NO – after activation of soluble guanylate cyclase – is the reversible inhibition upon binding to cytochrome oxidase (complex IV).

**Figure 5:**
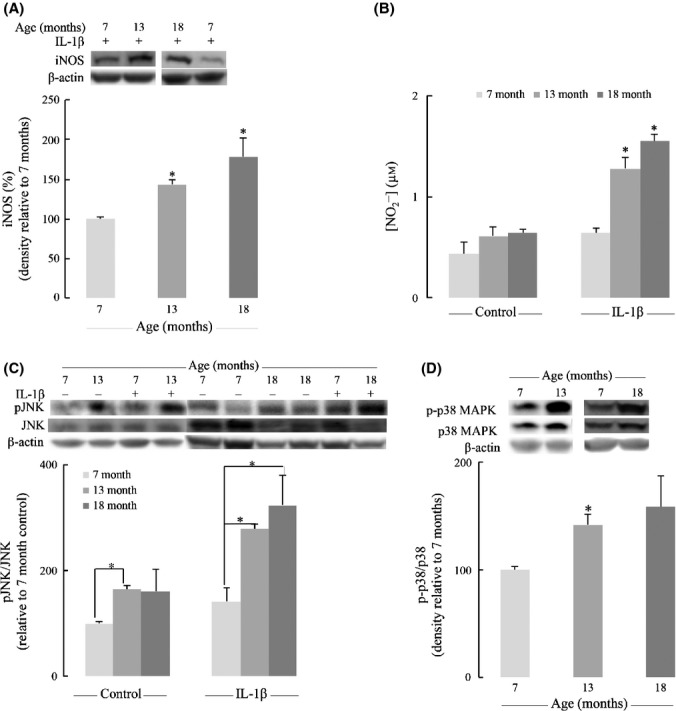
IL-1β induces activation of inducible nitric oxide synthase (iNOS) and JNK/p38 pathways in astrocytes – effect of age. (A) Astrocytic iNOS expression induced by 100 ng mL^−1^ IL-1β was determined by Western blot. (B) Nitrite in the medium of primary astrocytes was determined by DAN assay as described in the Experimental Procedures. (C) Effect of IL-1β (100 ng mL^−1^) on JNK activation (phosphorylation). (D) Age-dependent increase of MAPK p38 activation. *P <* *0.05.

The constitutive levels of JNK activation (pJNK/JNK values) were prominent in astrocytes from older rats: statistically significant higher pJNK/JNK values in astrocytes from older rats compared with those from 7-month-old rats. These values were increased upon treatment with IL-1β: pJNK/JNK values were 2.4- and 2.1-fold higher in astrocytes from 18- and 13-month-old rats than those in astrocytes from 7-month-old rats (Fig.5C). There was also an age-dependent activation of MAPK p38: p-p38/p38 values were substantially higher in astrocytes from 13-month-old and 18-month-old rats compared with astrocytes from young rats (Fig.5D).

Jun N-terminal kinase activation is apparently at odds with the reported age-dependent increase in rat brain and its impact on neuronal metabolism: Active (bisphosphorylated) JNK translocates to mitochondria where it is docked on the outer mitochondrial membrane; this triggered a phosphorylation cascade that resulted in inhibition of the pyruvate dehydrogenase complex (upon phosphorylation of the E_1α_ subunit) and the detrimental consequence on energy metabolism (Zhou *et al*., 2008, 2009; Jiang *et al*., 2013). However, these experiments were performed in primary cortical neurons and it does not seem to apply to astrocytes. Here, we demonstrated that JNK is activated in primary astrocytes upon IL-1β treatment, and there is increased JNK activity with age both constitutively and in the presence of inflammatory cytokines. Activated JNK could be involved in the degradation of IκB, thus enhancing NFκB signaling, and is also involved in the induction of proinflammatory cytokine genes coding for TNFα, IL-6, and others (Mehan *et al*., 2011). JNK and p38 but not ERK were upregulated upon treatment of rat astrocytes with IL-1β and TNFα (Thompson & Van Eldik, 2009).

### Cytokine treatment increased astrocytic aerobic metabolism

It is well established that aging is associated with increased release of inflammatory cytokines including IL-1β and TNFα. Data in Fig.2 indicate an age-dependent increase of mitochondrial oxidative metabolism in astrocytes, and data in Figs4 and 5 indicate that the total constitutive levels of NFκB increase in cytosol and nucleus and that translocation to the latter is strongly stimulated by the cytokines IL-1β and TNFα. Hence, the question as to the effect of cytokines on astrocyte metabolism (OCR) needs to be addressed: Primary cultured astrocytes treated with different concentrations IL-1β and TNFα showed dose-dependent increases in mitochondrial respiration (Fig.6). At the highest IL-1β concentration (1000 ng mL^−1^), basal respiration was increased 3.7-fold (from 45.7 pmoles min^−1^ in control to 171.4 in IL-1β-supplemented astrocytes) and the reserve capacity 2.2-fold (from 51.4 pmol min^−1^ in control to 114.2 in IL-1β-supplemented astrocytes) (Fig.6A). TNFα at the highest concentration (500 ng mL^−1^) exerted similar effects: 70% increase in basal respiration and a more robust (2.1-fold) increase in astrocytic reserve capacity (Fig.6B).

**Figure 6:**
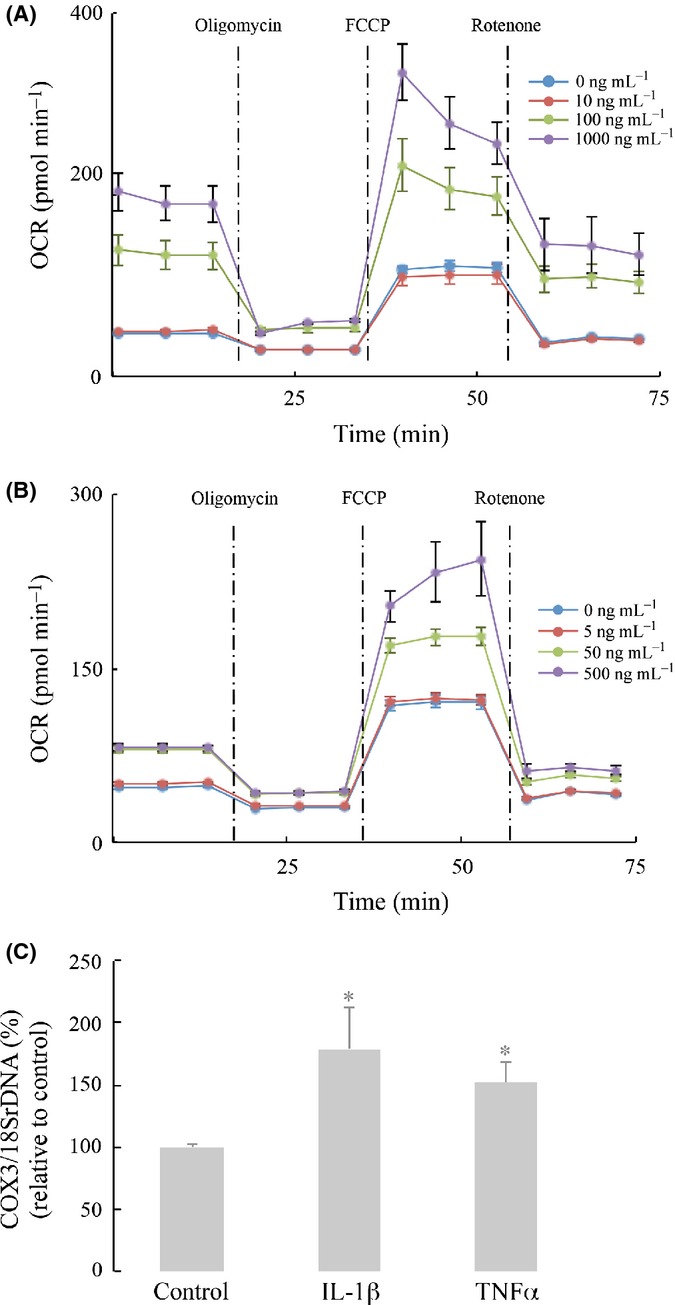
Cytokine treatment increased astrocytic aerobic metabolism. Primary astrocytes from 13-month-old rat brains were cultured on Seahorse XF-96 plates. Effects of (A) IL-1β and (B) TNFα on oxygen consumption rate (OCR). Different concentrations of IL-1β and TNFα are indicated in the figure. (C) Increases in mitochondrial biogenesis by inflammatory cytokines (IL-1β, 100 ng mL^−1^; TNFα, 50 ng mL^−1^) were determined by the ratio of COX3/18SrDNA assayed by real-time PCR. *P <* *0.05.

The effects of these cytokines on COX3/18SrDNA values (reflecting mitochondrial biogenesis) are shown in Fig.6C: IL-1β increased COX2/18SrDNA values ∼79% and TNFα ∼52%. This indicates that cytokines may enhance mitochondrial respiration (oxidative metabolism) by increasing mitochondria number.

## Discussion

Although microglia are generally considered the resident immune cells in the brain, the impact of astrocytes on inflammation cannot be understated. First, astrocytes outnumber microglia in the brain; second, astrocytes detect and amplify inflammatory signals from microglia, especially by self-propagation of the cytokine cycle to generate large amount of cytokines within a short period of time, and last but not least, astrocytes are in close proximity to neurons and synapses, so they directly affect neuronal functions.

A salient feature of this study is the age-dependent astrocytic metabolic shift: Astrocytes rely primarily on ATP derived from glycolysis and the final product, pyruvate, is reduced to lactate, which supports energy demands in neurons when released from astrocytes (Dienel & Hertz, 2001; Bolaños *et al*., 2010; Magistretti, 2011; Suzuki *et al*., 2011). In turn, astrocyte-derived lactate is oxidized to pyruvate in neurons, a substrate for the pyruvate dehydrogenase complex that furnishes acetyl-CoA to the tricarboxylic acid; the energy-carrying molecules in this case are the electron-rich NADH and FADH_2_. Because of this neurotrophic support of astrocytes, it is interesting that mitochondrial oxidative metabolism in astrocytes is enhanced with age, especially considering the general energy deficit or hypometabolic state associated with brain aging. However, few studies actually distinguished astrocytic metabolism from neuronal metabolism in aging. Furthermore, astrocytes mainly rely on anaerobic glycolysis for energy, and its oxidative metabolism is generally much weaker than that of neurons, so its alterations with age could be easily masked if mitochondrial metabolism measures are performed on the whole brain. Astrocytes are generally considered neurotrophic inasmuch as providing neurons with energy substrates and recycling neurotransmitters. However, data in this study showed that astrocytes reduced their neurotrophic functions by utilizing energy substrates for their own metabolism rather than transferring them to neurons. Hence, the high energy demands imposed by neuronal function are not supported by the age-declining neurotrophic function of astrocytes; challenges to this neurotrophic support – in terms of astrocyte-generated lactate – become exacerbated with the age-dependent reduction in expression and translocation to the plasma membrane of neuronal glucose transporters (GLUT3 and GLUT4) (Jiang *et al*., 2013).

An argument supporting this increase in ‘energy-efficient metabolism in astrocytes’ is that inflammatory reactions in astrocytes in response to infection or other stressors are metabolically expensive events (Johnson *et al*., 2012) and may stimulate mitochondrial metabolism for the energy support. This notion is support by the finding that IL-1β and TNFα stimulate mitochondrial metabolism, which is likely to be the scenario in aging too. Aging is usually accompanied by recurrent injury, invasion, and other insults causing chronic inflammation. Astrocytes amplify inflammatory signals rather than standing in the first line of defense as microglia; therefore, their sustained production of inflammatory mediators require a steady supply of ATP, which could be supported by oxidative metabolism rather than glycolysis. Increased mitochondrial metabolism was postulated to result from mitochondrial biogenesis, but not higher mitochondrial quality, and inflammatory cytokines were shown to induce mitochondrial biogenesis to support metabolic functions and cell viability (Piantadosi & Suliman, 2012). An increase in reactive astrocytes is a hallmark of brain aging and neurodegenerative diseases. It may be surmised that increased mitochondrial aerobic metabolism and inflammatory responses support the functionality switch of astrocytes, from neurotrophic to neurotoxic with age. Strategies that modulate substrate metabolism in astrocytes would be innovative approaches to address impaired neuronal function and neurodegeneration with age. In terms of redox status, astrocytes have a more reducing redox environment than that of neurons and are less sensitive to apoptosis: A good correlation was found between cell viability and the cell's redox potential (calculated from the cellular levels of GSH and GSSG). This was also reflected by the different sensitivity of neuronal and astrocytic glyceraldehyde-3-phosphate dehydrogenase (GAPDH) to S-nitrosylation and S-glutathionylation (Yap *et al*., 2010).

It has been shown that TNFα, IL-1β, and IL-6 expression increased in rat brain cortex and striatum during aging, and the expression of cytokines was mostly attributed to astrocytes, but not in microglia or neurons (Campuzano *et al*., 2009). NFκB is the master regulator in inflammation. The activation of canonical NFκB pathway follows binding of IL-1β and TNFα to their receptors, activation of IKK complex, degradation of IκBα, and translocation of cytosolic NFκB to the nucleus, where it initiates transcription of a set of inflammation-related genes. Activation of NFκB pathway along with MAPK was found to account for increased chemokines CCL2/MCP-1 and CCL7/MCP-3 in rat astrocytes treated with IL-1β and TNFα (Thompson & Van Eldik, 2009). Constitutive levels of NFκB in the cytosol were substantially increased in aging (Fig.4A), which serves as a free pool ready to enter nucleus and induce transcription of inflammatory genes. The most determining evidence for NFκB activation with age was the elevation of the constitutive level of NFκB in the nucleus (Fig.4C,D). However, the sensitivity of NFκB signaling to cytokines was increased in aging. Specifically, upon stimulation by IL-1β, young astrocytes showed moderate activation of cytosolic NFκB signaling (assessed by decreased IκBα/NFκB ratio), whereas astrocytes from old rats exhibited a much stronger response to IL-1β (Fig.4B). In support of this, upon IL-1β stimulation, older astrocytes had a larger increase in nuclear translocation of NFκB than young astrocytes. H_2_O_2_ has been shown to play an important role in NFκB activation and signaling (Oliveira-Marques *et al*., 2013), and this study suggests that the age-dependent increase in H_2_O_2_ generation by astrocytes is largely driven by an increased NOX2 expression, the NOX isoform typically induced by inflammation (Sorce & Krause, 2009) and slight increases in NOX4, the constitutive isoform. In this context, NADPH oxidases were elevated and activated in brains from patients with Alzheimer's disease and Parkinson's disease (Park *et al*., 2005). Activation of NOX enzymes in astrocytes exposed to toxic stimuli such as Amyloid β was found to induce neuronal damage (Abramov *et al*., 2004; Abramov & Duchen, 2005).

In this study experimental model, •NO is produced by iNOS in the astrocytes, which is regulated by NFκB at a transcriptional level. •NO is involved in the regulation of a broad spectrum of pathophysiological processes in the brain, with major impacts on signaling via cGMP, regulation of mitochondrial function (upon reversible binding to cytochrome oxidase), and multiple S-nitrosylation targets associated with cellular functional responses. •NO derived from astrocytes resulted in glia-induced neuronal death (Bal-Price & Brown, 2001). This study showed that •NO released from astrocytes increased substantially with age both constitutively and in the presence of inflammatory cytokines (Fig.5B). The increase in •NO production upon IL-1β stimulation is even more pronounced at older ages, which, in turn, resulted from the age-dependent increased sensitivity of NFκB signaling. It is conceivable that •NO is exported from astrocytes and elicit damage (in neurons) at sites distant from its generation. Although the elevated •NO levels by astrocytic iNOS may predict mitochondrial damage and cell toxicity, results from this study suggest that astrocytes may be resistant to •NO toxicity. Astrocytes were more resistant to •NO toxicity than neurons due to their elevated GSH synthesis (Gegg *et al*., 2003) and displayed a much lower sensitivity than neurons, when both exposed to a steady flux of •NO, due to a higher capacity to recover GSH through glutathione reductase (Yap *et al*., 2010). These arguments, however, cannot bridge the discrepancy between the age- and iNOS-dependent •NO generation (Fig.5A,B) and the age-dependent increase in mitochondrial oxidative metabolism (Fig.2). In this regard, it was reported that iNOS stimulated hepatic mitochondrial biogenesis by a mechanism entailing association of iNOS with the outer mitochondrial membrane, its binding and S-nitrosylation of Hsp60 and Hsp70, thus promoting Tfam accumulation in mitochondria (Suliman *et al*., 2010).

## Experimental procedures

### Isolation and culture of primary astrocytes

Primary astrocytes were isolated from brain cortices of 6–7-month-old, 12–13-month-old, and 18-month-old Sprague–Dawley male rats. Rats from different age groups were sacrificed on the same day, and brains were rapidly dissected in ice-old HBSS. Cortices were subject to Trypsin digestion and repetitive trituration to extract glia cells. Cells were then filtered and plated into poly-D-lysine coated flasks in Dulbecco's modified Eagle's medium/F12 culture medium supplemented with 20% fetal bovine serum, 0.5 U mL^−1^ penicillin, and 0.5 mg mL^−1^ streptomycin, with medium renewal every other day during the first week and 2 times a week starting second week. Once reached confluence after 2–3 weeks, cells were placed onto an orbital shaker for 4 h to shake off microglia and yield enriched astrocytes for experiments. Purity of the primary astrocyte culture was assessed by GFAP (an astrocyte specific intermediate filament) staining; OX-42 was used as a microglial marker (Morgan *et al*., 1995).

### Cytosolic and Nuclear Fraction isolation

Cytosolic and nuclear fractions were isolated from primary astrocytes using NE-PER™ Nuclear and Cytoplasmic Extraction Reagents (Pierce Biotechnology, Rockford, IL, USA) following manufacturer's instructions.

### DNA isolation and quantification

Total DNA from primary astrocytes was prepared using Wizard Genomic DNA Purification Kit (Promega Corporation, Madison, WI, USA) and following the manufacturer's instructions. The relative copy numbers of mitochondrial and nuclear DNA were determined by real-time PCR with primers specific to the COX3 (mitochondrial) and 18SrDNA (nuclear) genes, 100 ng DNA, and SYBRGreen PCR master mix (Bio-Rad, Hercules, CA, USA) on an iCycler real-time PCR machine (Bio-Rad).

### Metabolic flux analyses

Primary astrocytes were cultured on Seahorse XF-24 or Seahorse XF-96 (Seahorse BioSciences, Billerica, MA, USA) plates at a density of 75 000 cells per well and 20 000 cells per well, respectively. On the day of metabolic flux analysis, media was changed to Krebs-Henseleit buffer (KHB), pH 7.4, supplemented with 25 mm glucose and/or 1 mm pyruvate and incubated at 37 °C in a non-CO_2_ incubator for 1 h. All medium and injection reagents were adjusted to pH 7.4 on the day of assay. Using the Seahorse XF (Seahorse BioSciences) metabolic analyzer, three baseline measurements of oxygen consumption rate (OCR) were sampled prior to sequential injection of mitochondrial inhibitors. Three metabolic determinations were sampled following addition of each mitochondrial inhibitor prior to injection of the subsequent inhibitors. The mitochondrial inhibitors used were oligomycin (4 μm), FCCP (carbonyl cyanide 4-(trifluoromethoxy)- phenylhydrazone) (1 μm), and rotenone (1 μm). OCR was automatically calculated and recorded by the Seahorse software. After the assays, protein level was determined for each well to confirm equal cell density per well.

### Measurement of mitochondrial membrane potential

Cells were harvested, washed with PBS, and stained with 30 nm of TMRM for 30 min, then washed with PBS. TMRM signal, which measures mitochondrial membrane potential, was analyzed with a flow cytometer (BD Biosciences). Data were collected from 10 000 cells from each sample and analyzed with the software WinMDI.

### Measurement of H_2_O_2_ and nitrite

H_2_O_2_ generation and release rates by primary astrocytes were determined by the Amplex Red/Peroxidase Assay kit (Invitrogen, Carlsbad, CA, USA) following the manufacturer's instructions. NO production was measured as total 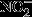 present in the medium and detected by DAN assay using Nitrate/Nitrite Fluorometric Assay Kit (Cayman Chemical, Ann Arbor, MI, USA).

### Western blot analysis

Cytosolic and nuclear fraction from primary astrocytes were solubilized in SDS sample buffer, separated by SDS/PAGE, and transferred onto PVDF membranes. Using appropriate antibodies, the immunoreactive bands were visualized with an enhanced chemiluminescence reagent. The blots were quantified using UN-SCAN-IT gel 6.1 (Silk Scientific, Inc., Orem, UT, USA).

### Statistical analysis

Data are reported as means ± SEM of at least 4–5 independent experiments. Significant differences between mean values were determined by Student's *t*-test.

## Author contributions

The experiments were designed by TJ and EC, and carried out by TJ. The manuscript was prepared by TJ and EC.

## Funding

Supported by NIH Grant RO1AG016718.

## Conflict of interest

None declared.
